# Sensitization to inhaled allergens in asthmatic children in southern Jordan: a cross-sectional study

**DOI:** 10.1186/s40248-019-0199-y

**Published:** 2019-11-08

**Authors:** Enas M Al-Zayadneh, Nedal Awad Alnawaiseh, Areej Hamed Altarawneh, Ibrahim Hamed Aldmour, Eman M. Albataineh, Hani Al-Shagahin, Abdelrahman Alharazneh, Ebaa Alzayadneh

**Affiliations:** 1 Department of Pediatrics, School of Medicine, University of Jordan, Amman, Jordan; 2 Department of Public Health, University of Mutah, Al-Karak, Jordan; 3 Karak Governmental Hospital, Ministry of Health, Al-Karak, Jordan; 4 Department of Microbiology and Immunology, University of Mutah, Al-Karak, Jordan; 5 Department of Special Surgery, University of Mutah, Al-Karak, Jordan; 6 Department of Physiology and Biochemistry, School of Medicine, The University of Jordan, Amman, Jordan

**Keywords:** Asthma, Inhaled allergens, SPT, Jordan

## Abstract

**Background:**

Sensitization to inhaled allergens in children with bronchial asthma significantly affects asthma pathogenesis, severity and persistence into late childhood and adulthood. The present study determined the prevalence of sensitization to inhaled allergens in children with bronchial asthma and wheezing episodes in order to investigate the effect of positive sensitization on the severity and control of asthma symptoms and to screen for other associated allergic conditions.

**Methods:**

A cross-sectional study was conducted, including children between 6 months and 14 years of age attending the chest clinic of Al-Karak, south of Jordan, between November 2013 and February 2016. Skin prick tests (SPTs) using 11 standardized allergen extracts were conducted in 277 children. The severity of asthma was determined based on the Global Initiative for Asthma (GINA) assessment and the Childhood Asthma Control Test (C-ACT) in addition to the history of use of systemic steroids and hospital admissions within the past 12 months.

**Results:**

Sixty-seven percent of children with bronchial asthma reported sensitization to one or more of the inhaled allergens. The most common allergens were olive pollens (18%), cat fur (13.5%), and *Dermatophagoides pteronyssinus* (11.9%). There was a significant increase in allergen sensitization with age (*p* < 0.001). The most common concomitant allergic condition among children was allergic rhinitis (71.5%); however, allergic conjunctivitis was the only allergic condition that correlated with the skin test reactivity (*p* = 0.01). A family history of bronchial asthma was confirmed in 40.4% of children. Children with positive SPTs had lower ACT scores and reported more frequent use of systemic steroids and admissions to hospital within the past 12 months; however, this effect was not statistically significant (*p* > 0.05).

**Conclusions:**

Sensitization to inhaled allergens is highly prevalent in children with asthma and wheezing episodes in southern Jordan and may be correlated with the severity of the disease. Therefore, appropriate measures to recognize and avoid these allergens are highly recommended. Most children in our study suffered from concomitant allergic rhinitis, indicating that an appropriate diagnosis and treatment of allergic rhinitis could significantly improve asthma control and thus the quality of life of these children.

**Trial registration:**

This study is not a clinical trial.

## Background

Bronchial asthma is one of the most common chronic disorders in children, and it imposes a great burden on global health. It is a major cause of frequent admissions to hospital and emergency room visits among the pediatric population, leading to significant morbidity and mortality in children worldwide [1]. Thus, bronchial asthma has a substantial impact on the health and quality of life of children as well as the economy [2–4].

In Jordan, it is estimated that 10% of the population has been diagnosed with bronchial asthma. According to an official report of the Jordanian Ministry of Health, most of these patients are children under the age of 15 years. One study estimated the prevalence of bronchial asthma in children in the capital city and rural northern Jordan to be approximately 9% [4]. Although the prevalence of asthma among school children was found to be 3% in the city of Al-Karak, southern Jordan, up to 16% children have had one or more wheezing episodes in their lifetime [5]. In the latter study, 36% reported symptoms of at least one allergic condition. Prevalence studies on childhood asthma at a national level are still lacking.

Bronchial asthma is caused by a complicated interaction between several environmental and genetic factors. In general, the triggering factors of asthma are classified as allergic (atopic) and non-allergic [6]. Atopy is defined as an individual’s intrinsic propensity to generate immunoglobulin E (IgE) antibodies in response to an exposure to certain allergens, with a higher tendency for developing typical allergic diseases, such as asthma, rhinitis, conjunctivitis, and atopic dermatitis. Generally, atopy is confirmed by the presence of serum allergen-specific IgE antibodies or positive skin prick tests (SPTs) [6]. Atopic asthma is the most common form of asthma in the pediatric population and is distinguished by the presence of eosinophils in the airways associated with specific IgE antibodies to several allergens, as verified by serology or SPT [7].

Sensitization to at least one inhaled allergen in atopic children is a prognostic and predictive indicator for developing asthma and for the persistence of asthma into late childhood and adulthood [8, 9]. For example, a previous study reported 50 to 95% sensitization to inhaled allergens in several patients with bronchial asthma [10]. Allergens trigger asthma attacks in 60 to 90% of atopic children and in 50% of atopic adults [11–13]. These inhaled allergens include seasonal pollen, mold spores, dust mites, and animal dander allergens. Identifying sensitization to inhaled allergens in children with asthma can assist in planning an environmental control strategy, titration of therapy (i.e., seasonal exacerbations), or referring patients for an immunotherapy regimen [13–15].

The present study determined the prevalence in Al-Karak of sensitization to inhaled allergens in children with bronchial asthma and wheezing episodes to investigate the effect of positive sensitization on the severity and control of asthma symptoms and to screen for other associated allergic conditions.

## Methods

### Study design and setting

We conducted a 2-year, cross-sectional study in the chest clinic at the Al-Karak Governmental Hospital. This clinic is the only pediatric chest clinic in Al-Karak and treats approximately 800 to 1000 children annually. Two hundred seventy-seven children aged between 6 months and 14 years were included in the study. These children were diagnosed with asthma or recurrent wheezing episodes according to the Global Initiative for Asthma (GINA) criteria: a physician’s diagnosis of asthma; symptoms of recurrent episodes (i.e., more than two) of wheezing, cough, shortness of breath, or a combination of these; documented reversibility of these episodes with bronchodilators; and the use of medication for asthma during the previous 6 months [13, 16].

### Skin prick test

A skin prick test was performed to identify sensitization to inhaled allergens. The test has a sensitivity of 80 to 97% and a specificity of 70 to 95%. SPT was performed using 11 standardized allergen extracts provided in a commercial test kit (Stallergenes Greer; Antony, France) in accordance with the published guidelines [17]. The 11 tested inhaled allergens included cat pelt, salsola-kali, *Dermatophagoides pteronyssinus* (*D. pteronyssinus*), and *Dermatophagoides farinae* (*D. farinae*) (two species of house dust mites, cereal-mix, olive pollen, grass mix, mold (*Alternaria*), dog fur, and composite- and wall-pellitory (weed pollens). A positive and a negative control (histamine and normal saline, respectively) were used to avoid false-negative results, e.g., patient consumption of antihistamines, or false-positive results, e.g., dermatographism.

To perform the SPT, allergen extracts were injected intradermally into each patient’s forearm, back, or shin. The results were interpreted 15 min after the injection. The test was reported to be positive if the prick site swelled up to form a wheal with a diameter greater than 4 mm. Parents and caregivers of the tested children were advised to stop systemic antihistamines or leukotriene modifiers 4 to 5 days prior to testing.

### Demographic and clinical data collection

Data were collected from parents through face-to-face interviews. Detailed medical histories were obtained and physical examinations were performed on all patients. Patients were asked to fill out questionnaires to collect data on the patients’ age, sex, family history of asthma, use of systemic steroids, hospital admissions over the past 12 months, and the presence of other allergic conditions. Other concurrent allergic conditions that children included in the study were screened for included allergic rhinitis, atopic dermatitis or eczema, allergic conjunctivitis, and food allergies.

### Assessment of asthma severity

Asthma severity and control of symptoms in the study population were classified according to the GINA classification [18]. Additionally, a validated questionnaire, the Asthma Control Test (ACT), was used for children 12 years and older. Childhood ACT (C-ACT) was used for children between 4 and 11 years of age. ACT or C-ACT assess general asthma symptoms, the frequency of shortness of breath, use of inhalers, and asthma influence on the child’s functional status. Children are categorized as having controlled asthma (score more than 19) or poorly controlled asthma (score that equals 19 or less) [19], (https://www.asthma.com/additional-resources/asthma-control-test.html). Moreover, the severity of asthma was assessed by reporting the use of systemic steroids and admission to hospital due to asthma within the past 12 months. Parents of all children provided consent for participation in the study. The study was approved by the University of Mutah Ethics Committee. Procedures were followed in accordance with the Helsinki Declaration of 1975.

### Statistical analysis

Data are expressed as the means and standard deviations (SDs) for continuous variables and frequencies and percentages for categorical variables. The relevant demographic and clinical characteristics of the SPT-positive patients were compared with those of the SPT-negative patients using the χ^2^ test. Multiple logistic regression was used to investigate the possible predictors of SPT reactivity. Statistical analysis was performed using SPSS 21 (IBM; Armonk, NY, United States of America; 2012). The results were considered significant if *p* ≤ 0.05.

## Results

**Fig. 1:**
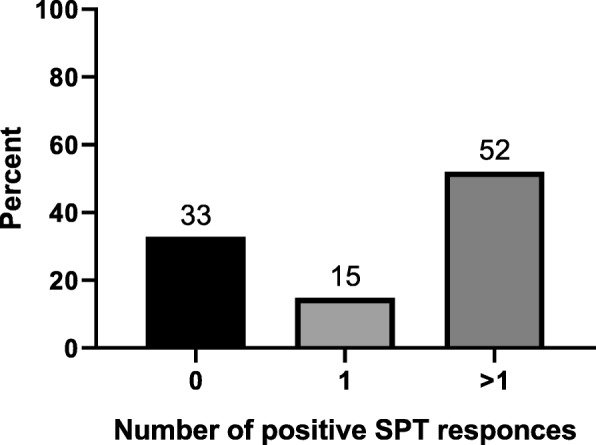
Frequency of negative and positive SPT in study children. SPT results are shown as (0) for negative SPT, (1) for positive SPT for one allergen, (< 1) for multiple allergens. Percent value is percentage of children who tested negative or positive (with respect to the number of positive responses) among all children of the study

**Fig. 2:**
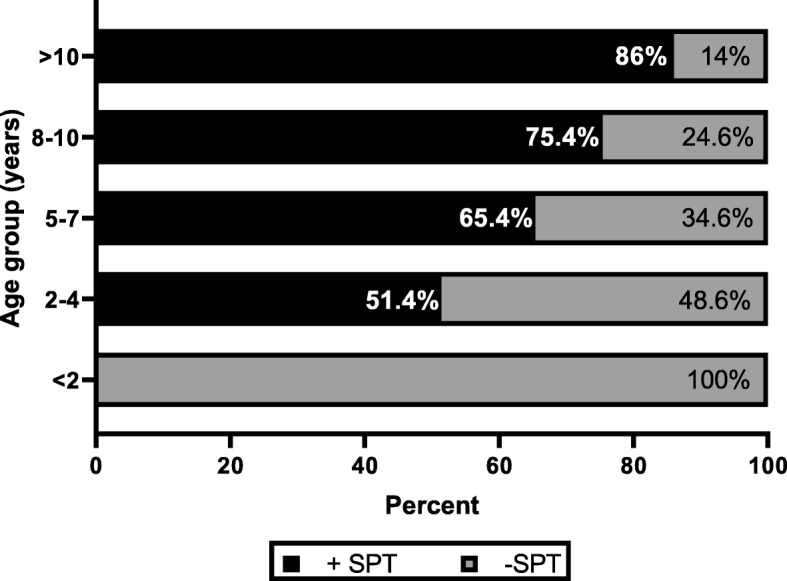
Age-group and sensitization to inhaled allergens. Positive SPT significantly increase with age (*P* = 0.001)

The present study included a total of 277 children with asthma and wheezing episodes. Demographics, GINA classification, and clinical profile are shown in Table 1. Of these 277 children, 186 (67%) had a positive SPT, 41 (15%) for a single allergen and 145 (52%) for multiple allergens (Fig. 1). A significant increase in the sensitivity with age was observed upon comparing SPT results among different age groups (χ^2^ = 24.7, *p* < 0.001) (Fig. 2). The maximum frequency of sensitivity was 86% in the group aged 10 years or older (Fig. 2).

**Table 1:** Demographic data and Clinical profile of study children

Children of study demographics	
Males	185 (66.7%)
Females	92 (33%)
Age: Mean ± SD	7.27 ± 3.37
Age groups:	
< 2	3 (1.1%)
2–4	74 (26.7%)
5–7	81 (29.2%)
8–10	69 (24.9%)
> 10	50 (18.1%)
Classification of asthma (GINA)	
Intermittent	115 (41.5%)
Mild persistent	74 (26.7%)
Moderate persistent	70 (25.3%)
Severe persistent	18 (6.5%)
Concomitant allergic conditions	
Allergic rhinitis	198 (71.5%)
Eczema	73 (26.4%)
Allergic conjunctivitis	83 (30.0%)
Food allergy	14 (5%)
Family History	
Asthma	112 (40.4%)

**Fig. 3:**
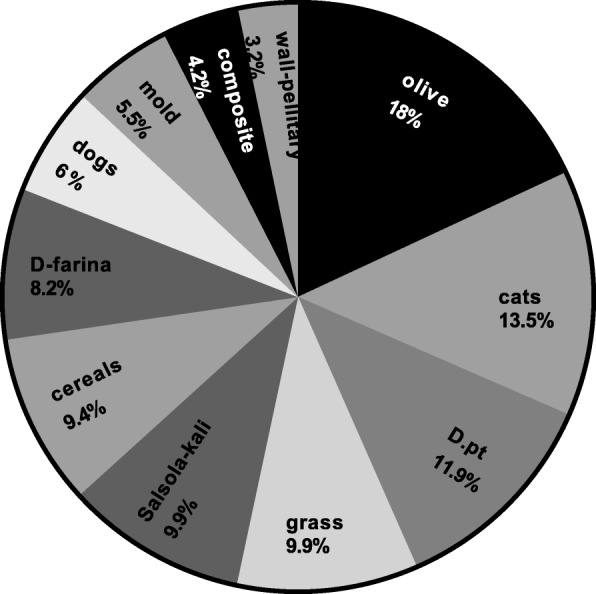
Frequency of sensitization to all tested allergens. % indicates percentage of children who tested positive to the respective allergen among children of the study

**Fig. 4:**
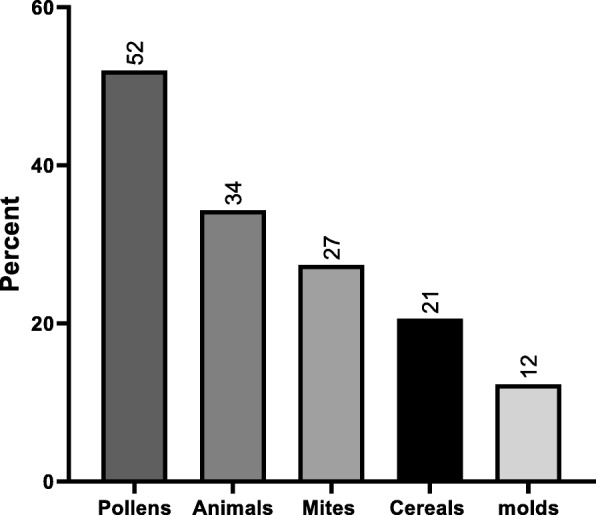
Frequency of sensitization to different groups of allergens. Percent indicates percentage of children who tested positive to the respective allergen group among children of the study. Allergens groups are as following: Pollens (Olive, Salsola-kali, grass mix, Wall-pellitory), Animals (Cat pelt and Dog fur), Mites (D.Pt, D.Farinae), Cereals, and molds (alternarea)

**Fig. 5:**
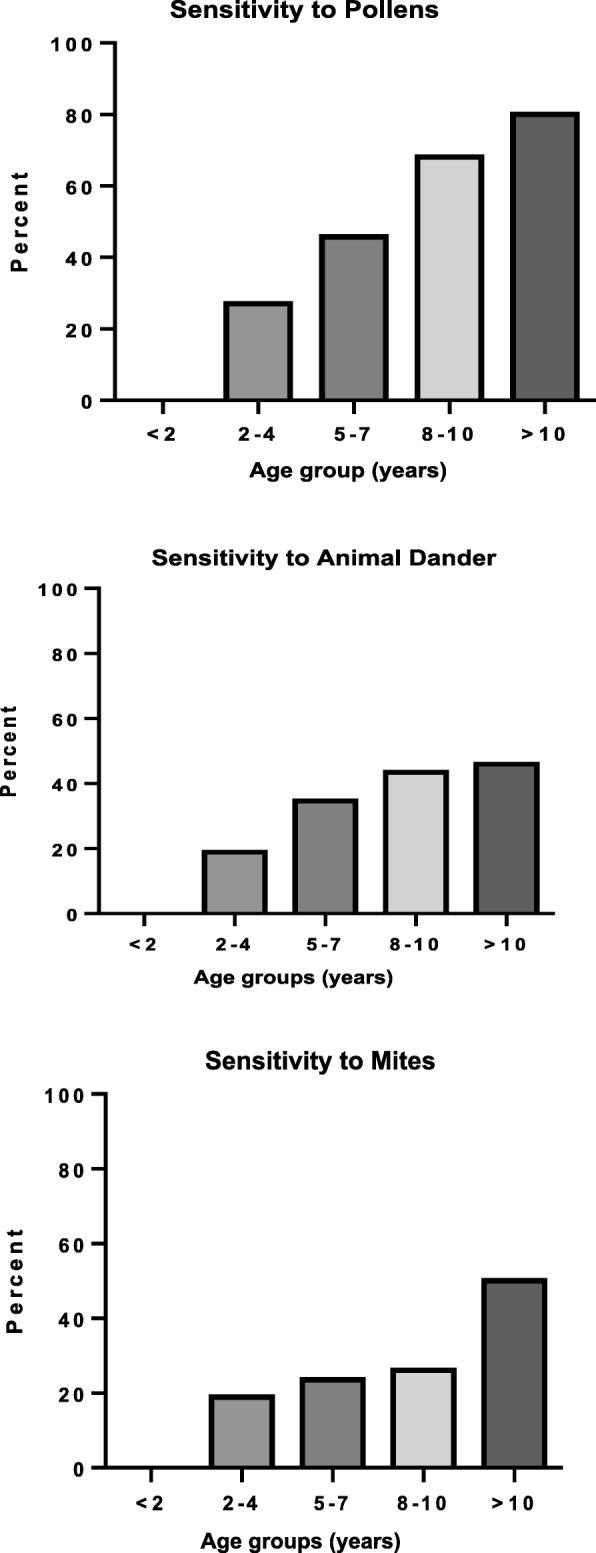
Sensitivity to Pollens, Animal dander and Mites among age groups

**Fig. 6:**
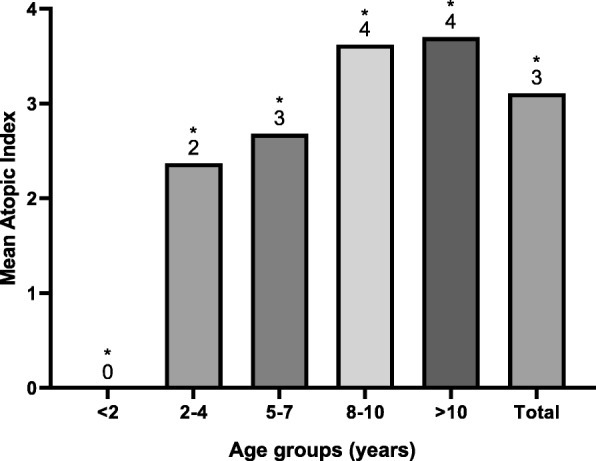
Mean atopic index (AI) for each age group. AI represents the total number of allergens for which the subjects had positive responses. (f-test = 7.8 and *p* < 0.010)

Figure 3 represents the frequency of sensitization for each allergen tested. Olive pollen had the highest frequency of sensitization (18%) followed by cat fur (13.5%) and *D. pteronyssinus* (11.9%). The sensitivity of patients to different groups of allergens (pollens, animal dander, mites, cereals, and molds) is described in Fig. 4. A skin reactivity to pollens was found in half of the children (144; 52%), with olive pollen causing the most common positive reaction (50; 18%), as shown in Figs. 3 and 4. There was a significant increase in the sensitivity with age to pollens (χ^2^ = 45.9, *p* < 0.001), animal dander (χ^2^ = 14.95, *p* = 0.005), and mites (χ^2^ = 17.32, *p* = 0.002), as shown in Fig. 5. The mean atopic index (AI) for each allergen group is shown in Fig. 6. The AI represents the total number of allergens out of the 11 tested to which the subjects exhibited positive responses. There was a significant difference in the mean AI among all age groups (*f*-test = 7.8 and *p* < 0.010).

Of the total number of children included in the study, 185 (66.8%) were boys and 92 (33.2%) were girls. No significant difference in the positive SPT responses was noted between boys and girls (68 and 65%, respectively; Table 2). Nevertheless, more boys were reported to be asthmatic with atopy compared to girls (126 boys/60 girls) in the present study (Table 2). According to the GINA classification of bronchial asthma, most of the children had intermittent asthma (115; 41.5%), followed by mild persistent (74; 26.7%) and moderate persistent asthma (70; 25%) (Table 1).

**Table 2:** Comparison between positive skin test and negative skin test children

	Positive Skin Test	Negative Skin Test
Males	126 (68.1%)	59 (31.9%)
Females	60 (65.2%)	32 (34.8%)
Allergic Conjunctivitis*	65 (78.3%)	18 (21.7%)
Eczema	51 (69%)	22 (30%)
Allergic Rhinitis	135 (68.2)	63 (31.8%)
Systemic Steroids	116 (67.4%)	56 (32.6%)
Family History of Asthma:	77 (68.8%)	35 (31.2)
GINA^a^ Classification:		
Intermittent	74 (64.35%)	41 (35.7%)
Mild persistent	48 (64.9%)	26 (35.1%)
Moderate Persistent	53 (75.7%)	17 (24.3%)
Severe Persistent	11 (61.1%)	7 (38.9%)
History of admission to hospital	104 (66.2%)	53 (33.8%)
Asthma control test score	N, Mean ± EM	#, Mean ± EM
	181, 16.5 ± 0.39	85, 17.08 ± 0.54

^a^*GINA* Global Initiative for Asthma, http://ginasthma.org

*×2 = 6.58, *p*-value = 0.01

N: Number of children

EM: Error of Measurement

The most common concomitant allergic condition among children was allergic rhinitis (198; 71.5%), followed by allergic conjunctivitis (83; 30%) and eczema (73; 26%) (Table 1). A positive SPT response was significantly higher among children who had concomitant conjunctivitis (65; 78.3%) (χ^2^ = 6.58, *p* = 0.01). However, there were no significant differences in the SPT reactivity among children with concomitant allergic rhinitis, atopic dermatitis and eczema, or food allergies (Table 2). A family history of bronchial asthma was reported in 112 children (40%) as shown in Table 1. Seventy-seven (68.8%) children with a family history of asthma reported a positive SPT; however, this was not predictive of SPT reactivity (Table 2).

Based on the results obtained using multiple logistic regression, we concluded that only age and concomitant allergic conjunctivitis were significant predictors of sensitization to inhaled allergens.

When children with positive SPT were assessed for severity according to the GINA classification, most asthmatic children above the age of 4 years (*n* = 170) were found to have intermittent asthma (40%), followed by mild persistent (25.9%) and moderate persistent asthma (28.9%), and only 5.3% of them had severe persistent asthma (Table 2). These findings were not significantly different when compared to the SPT-negative group (*p* > 0.05). One hundred four children (66.25%) were admitted to the hospital with an asthma exacerbation during the past 12 months compared to 53 (33.8%) children with a negative SPT. Systemic steroids were used to treat acute exacerbations in 116 (67%) children with a positive SPT compared to 56 (32.3%) children with a negative SPT. Neither admission to hospital nor use of systemic steroids was significantly different between positive-SPT and-negative SPT children (*p* > 0.05).

In addition, the mean ACT score was less than 19 (poor asthma control) in both SPT-positive and SPT-negative groups with no significant difference between the two groups (16.5 ± 5 and 17.08 ± 5), respectively (*p* > 0.05) (Table 2).

## Discussion

The present study found that sensitization to inhaled allergens is present in almost two-thirds of children with bronchial asthma and wheezing episodes. These findings are consistent with the results of previous studies [20–23]. Most children with a positive SPT were sensitive to multiple inhaled allergens, similar to the findings in other reports [24, 25]. Children with negative skin reactivity to SPT may lack atopy or be sensitized to other allergens not tested in this study. We analyzed the clinical and demographic characteristics of these children to identify possible predictive values for a positive SPT and found only age and concomitant allergic conjunctivitis to be significant predictors. Older children reported more positive SPT results for different groups of allergens (pollens, mites, and animal dander). This finding might be attributable to the natural history of atopic disorders (the allergic march), where exposure to indoor allergens occurs earlier in childhood and exposure to outdoor allergens, particularly tree pollens and grasses, occurs later and increases with age [26]. Furthermore, this may suggest that asthma in children with atopy is more likely to persist into later childhood and adulthood than non-atopic asthma. Covar et al. followed children in the Childhood Asthma Management Program study for 4 years and found that remission was associated with a lack of atopic sensitization [27].

The incidence of asthma and atopy in boys was higher, a finding consistent with a previous report [28]. However, there was no significant difference in SPT reactivity between boys and girls. In accordance with our findings, a study from Jordan that evaluated skin reactivity to 18 inhaled allergens in patients with allergic rhinitis found that most patients had sensitization to more than one inhaled allergen [29]. In the latter study, pollens of a grass mix, thistle weed, and olive tree were the most common seasonal allergens, while cat allergen was the most common perennial allergen, followed by house dust mites (*D. pteronyssinus*). These findings are consistent with our study, showing that olive tree pollen, cat fur allergen, and house dust mites (*D. pt*) were the most common inhaled allergens in children from Al-Karak.

In the present study, allergic rhinitis was found to be the most common allergic condition associated with bronchial asthma, occurring in approximately two-thirds of children. These findings are similar to those of previous reports [30] and emphasize the importance of screening all children with bronchial asthma for allergic rhinitis to determine the appropriate treatment for both conditions, thereby improving symptom control [30]. Although allergic rhinitis failed to show a predictive value for skin prick reactivity in our study, concomitant allergic conjunctivitis was a predictive variable for positive SPT. Several of the children included in the present study with positive sensitization also had a positive family history for one or more allergic conditions, indicating a genetic predisposition to asthma and atopy [9]. However, a family history of asthma was not predictive of skin test reactivity, which could be explained by the complexity of the interrelationship between the genetics and epigenetics of asthma [31].

As for the relationship between allergen sensitization and asthma severity, many studies have demonstrated that the presence of atopy has a linear relationship with asthma morbidity and that sensitization to allergens can be used as a marker for asthma severity [32–36]. Allergen sensitization to molds, cockroach, pet dander, and inhaled food-derived allergens has been reported to be associated with severe asthma episodes [37]. Exposure to environmental molds may lead to life threatening asthma episodes [38–40]. In addition, “thunderstorm asthma” was shown to be associated with severe symptoms resulting from massive and abrupt exposure to inhaled allergens [41].

However, the findings of the current study do not support this relationship. There was no statistically significant difference between SPT-positive and SPT-negative asthmatic children in terms of GINA classification, ACT score, admission to hospital or the use of systemic steroids within the past 12 months. This could be explained, in part, by the lack of more objective measures to assess the severity of asthma (e.g., spirometry for forced expiratory volume in the first second [FEV1]) and atopy (e.g., quantitative serum IgE level). In addition, most children in this study presented to the clinic with their care givers when their asthma symptoms were uncontrolled, rather than for a regular follow-up, which resulted in low clinical scores. Moreover, our allergen panel did not include some important allergens like cockroaches and various types of fungi (e.g., Aspergillus spp), which can be strongly associated with severe asthma in addition to house dust mites and seasonal pollens.

## Conclusions

Atopy is considered a significant burden in regards to childhood asthma. Most children with asthma and wheezy episodes in the present study had sensitization to one or more inhaled allergens. The two strains of house dust mites, olive pollen, and cat fur were the most common inhaled allergens. Age had a predictive value for allergen sensitization, as older children reported more positive SPT results. Allergic conjunctivitis was the only concomitant allergic condition that correlated with skin test reactivity. With regard to allergen sensitization and asthma severity, we could not demonstrate a significant relationship. Based on the findings of the present study, we concluded that allergen sensitization is highly prevalent in children with asthma and wheezing episodes. Therefore, physicians should be encouraged to perform allergen testing on all children with asthma. Additionally, families should be instructed to avoid allergens when possible to limit exposure of their atopic children to asthma triggers. The association of asthma with other allergic conditions, especially allergic rhinitis and conjunctivitis, is common and medical personnel should address these conditions during evaluation and treatment. Researchers are advised to conduct further studies with a larger population size including patients from other parts of Jordan to assess the prevalence and pattern of sensitization in children with bronchial asthma in order to improve asthma control and the quality of life of these children.

## Data Availability

The data are available from the corresponding author on request (einas_md@yahoo.com).

## Abbreviations

ACT: Asthma control test
AI: Atopic index
C-ACT: Childhood ACT
GINA: Global Initiative for Asthma
IgE: Immunoglobulin E
SPT: Skin prick test
